# Playing-Related Musculoskeletal Disorders, Risk Factors, and Treatment Efficacy in a Large Sample of Oboists

**DOI:** 10.3389/fpsyg.2021.772357

**Published:** 2022-01-14

**Authors:** Heather M. Macdonald, Stéphanie K. Lavigne, Andrew E. Reineberg, Michael H. Thaut

**Affiliations:** ^1^Faculty of Music, University of Toronto, Toronto, ON, Canada; ^2^Music and Health Science Research Collaboratory, Faculty of Music, University of Toronto, Toronto, ON, Canada; ^3^Institute for Behavioral Genetics, University of Colorado Boulder, Boulder, CO, United States; ^4^Faculty of Medicine, University of Toronto, Toronto, ON, Canada

**Keywords:** oboe, musculoskeletal disorders, woodwind, occupational health, prevalence, risk factors, musician, playing-related injuries

## Abstract

**Objectives:**

During their lifetimes, a majority of musicians experience playing-related musculoskeletal disorders (PRMD). PRMD prevalence is tied to instrument choice, yet most studies examine heterogeneous groups of musicians, leaving some high-risk groups such as oboists understudied. This paper aims to (1) ascertain the prevalence and nature of PRMDs in oboists, (2) determine relevant risk factors, and (3) evaluate the efficacy of treatment methods in preventing and remedying injuries in oboe players.

**Methods:**

A 10-question online questionnaire on PRMDs and their treatments was completed by 223 oboists. PRMDs were compared across gender, weekly playing hours, career level, age, and years of playing experience.

**Results:**

Of all respondents, 74.9% (167/223) reported having had at least one PRMD in their lifetime. A majority of these injuries (61.9% of all respondents) were of moderate to extreme severity (5 or higher on a scale of 1 to 10). Females (mean = 5.88) reported significantly more severe injuries than males. No significant effects of career level (i.e., professional vs. student vs. amateur), age, or years of playing experience were observed. We found significant non-linear relationships between weekly playing hours and PRMD prevalence and severity. Injuries were most commonly on the right side of the body, with the right thumb, wrist, hand, and forearm being most affected in frequency and severity. Of those injuries for which recovery information was provided, only 26.1% of injuries were “completely recovered.” The perceived effectiveness of a few treatments (physical therapy, rest, stretching, occupational therapy, massage) tended to be ranked more highly than others.

**Conclusion:**

The oboists in this study experienced high rates of PRMD, particularly in the right upper extremities. Females and those playing 7-9 and 16-18 h per week reported a significantly higher severity of injuries than other groups.

## Introduction

At some point in their careers, most musicians experience the painful and disabling effects of playing-related musculoskeletal disorders (PRMD). For professional musicians, these injuries can mean lost income and increased financial precarity, social stigma and strain on relationships, and the psychological and emotional hardships of not being able to make music (Guptill, 2011).

Research has provided insight into the nature and prevalence of PRMD in musicians in general; however, the majority of these studies deal with large, heterogeneous groups of musicians, leaving some specific groups of musicians understudied. Preliminary findings suggest that one of these understudied groups, oboists, are among the most at risk (Thrasher and Chesky, 2001; Nemoto and Arino, 2007; Stanek et al., 2017). This elevated risk is most often attributed to the oboe’s asymmetrical playing position (Thrasher and Chesky, 2001; Nemoto and Arino, 2007). Professional-level oboes weigh between 590 and 735 grams (J. Mason, personal communication, November 13, 2020), and this entire weight is supported by the right thumb, leading to possible strain and injury in the right upper extremities. Despite their elevated risk, oboists are typically underrepresented in studies (three largest sample sizes: *n* = 60, 28, and 12) (Thrasher and Chesky, 2001; Nemoto and Arino, 2007; Stanek et al., 2017), and there is little consensus on the precise nature and prevalence of PRMD in this population. To gain a fuller and more nuanced understanding of oboists’ PRMD, researchers must target a larger sample size of oboe players. In this paper, we present the results of the first large-scale survey of oboists designed to capture variation across the spectrum of career level, experience, age, and gender.

Although much work has been done to identify PRMD risk factors in musicians in general, little is known about the extent to which these risk factors hold true for oboists. Risk factors identified in musicians at large include time spent playing the instrument (Fry, 1987; Manchester and Park, 1996; Ranelli et al., 2011; Fotiadis et al., 2013; Lopez and Martinez, 2013; Robitaille et al., 2018), and younger age (Yeung et al., 1999; Davies and Mangion, 2002; Ranelli et al., 2011; Nawrocka et al., 2014; Kok et al., 2018; Selms et al., 2020). Due to small sample sizes of oboists in existing studies, it has not yet been established to what extent these factors influence PRMD prevalence in oboe players. Only one study to date has had a large enough sample size to infer anything about demographic predictors of PRMD in oboists, yet this study only examined the effects of gender (females were more likely to be injured than males), and did not collect data on the many other possible demographic risk factors (Thrasher and Chesky, 2001).

Similarly, studies on treatment methods have typically tested interventional exercise or postural methods on heterogeneous groups of musicians in which the oboe is underrepresented (Brandfonbrener, 1997; de Greef et al., 2003; Lopez and Martinez, 2013; Chan et al., 2014; Arnason et al., 2018). While some of these methods showed promise, others were inconclusive, often due to a low participation or high dropout rate. This search for a “one size fits all” cure for PRMD ignores the diversity of physical challenges presented by various instruments. Since injury rates and types are tied to instrument choice, it follows that treatment methods will have more consistent results when evaluated in a group of instruments with the same or similar issues.

To simultaneously address all the issues pointed out above, this survey has three main aims. (1) We aimed to ascertain the prevalence and nature of PRMDs in oboists. We anticipated that rates would fall within or near the 72-85.7% range established by previous studies with smaller sample sizes of oboists, and that injuries would be primarily on the right side of the body. (2) We also aimed to determine any predicting demographic or other risk factors. Based on existing literature, we hypothesized that female gender, a greater number of playing hours, and younger age or fewer years of experience would all be associated with higher rates of injury. Few existing studies evaluated career level (e.g., student vs. professional vs. amateur) as a risk factor, but based on our understanding of the demands of these various career levels, we anticipated that professionals and students would experience a higher rate of injury than amateurs, who are more likely to be playing for fewer hours per week. (3) Lastly, we aimed to evaluate the efficacy of various treatment methods in preventing and remedying these injuries in oboe players. We anticipated perceived effectiveness of the methods listed would have high variability.

## Materials and Methods

This project obtained approval from the University of Toronto Research Ethics Boards (protocol #39562). Participants were recruited via email and oboe-specific Facebook groups between September 3rd and September 21st, 2020 to complete a voluntary 10-question survey on their history with playing-related musculoskeletal disorders and treatments.

### Participants

**FIGURE 1:**
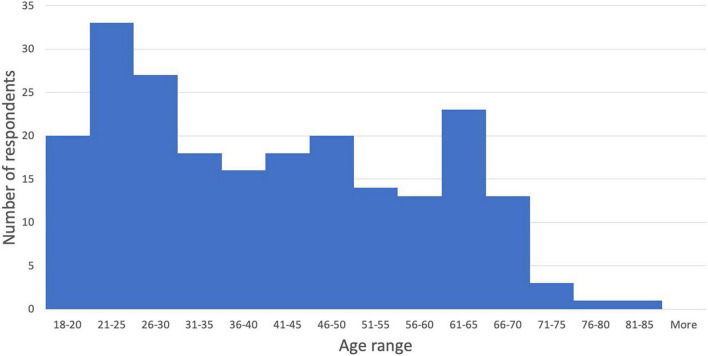
Age distribution of sample.

**FIGURE 2:**
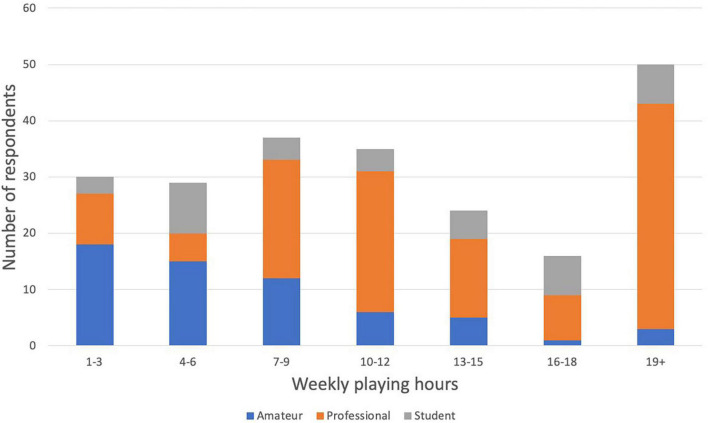
Weekly playing hours responses by career level.

Participants were 223 self-identified oboists recruited from oboe and double-reed Facebook groups (primarily English-speaking) and from emails to oboe professors at North American universities. The average age of the sample was 41.1 (Range = 18 - 81; *SD* = 16.50) (see Figure 1). Three respondents did not specify age. A large majority of respondents identified their gender as female (70.0%, *n* = 156). Other responses for gender were male (28.1%, *n* = 65), non-binary (0.4%, *n* = 1), and other (0.4%, *n* = 1, who specified in the comments being a trans man). Participants were diverse in professional status: 39 students (17.5%), 61 amateurs (27.4%), and 123 professional (55.2%). Participants had an average of 27.69 years of experience on oboe (Range = 0 - 69; *SD* = 16.96). The average age at which they started oboe was 13.37 (range = 3 - 62; *SD* = 7.04). The weekly time spent playing the oboe (see Figure 2) was reported as 1-3 h (13.6%, *n* = 30), 4-6 h (13.1%, *n* = 29), 7-9 h (16.7%, *n* = 37), 10-12 h (15.8%, *n* = 35), 13-15 h (10.9%, *n* = 24), 16-18 h (7.2%, *n* = 16), and 19+ h (22.6%, *n* = 50). 2 respondents did not specify weekly playing time.

### Survey Materials and Procedure

The 10-question survey was conducted online using the SurveyMonkey platform. The survey took an estimated 5-7 min to complete. Three existing questionnaires were consulted in the making of this survey (Kuorinka et al., 1987; Ackermann and Driscoll, 2010; Lamontagne and Belanger, 2012). Ultimately, most questions were adapted from Ackermann and Driscoll (2010) because of its specific tailoring to musicians and the applicability of its questions to our research questions. The first 5 questions collected demographic data (age, gender, oboe start age, career level, weekly playing time), and the latter 5 questions asked about respondents’ experiences with injury and preventive methods. As in Ackermann & Driscoll’s questionnaire, a playing-related musculoskeletal disorder (PRMD) was defined as “any pain, weakness, numbness, tingling, or other symptoms that interfere with your ability to play your instrument at the level to which you are accustomed,” which itself was based on the definition used by Zaza and Farewell (1997). Respondents were asked to identify any PRMDs they had experienced, from a list of 22 specified body parts plus “other (please specify),” and to rate the severity of the injury on a scale of 1 (mild) to 10 (extremely severe). The list of injury locations was adapted from two surveys (Kuorinka et al., 1987; Ackermann and Driscoll, 2010). Two main changes were made to the lists: since the majority of PRMDs reported to date among woodwind players occur in the upper extremities (Nemoto and Arino, 2007), the list of upper extremity injury locations would ideally be more nuanced than that in the general purpose Nordic Musculoskeletal Questionnaire (NMQ; Kuorinka et al., 1987). Therefore, instead of broader categories such as “wrist/hand,” the upper extremities were subdivided into fingers, thumb, hand, wrist, forearm, elbow, upper arm, and shoulder as in Ackermann and Driscoll (2010). Furthermore, since injuries in the lower body (feet/ankles, legs, knees) are unlikely to be caused by or to have a strong effect on playing a wind instrument, these injury locations were omitted and replaced with “other (please specify).” The 1 to 10 or 0 to 10 scale for rating pain and effectiveness has been used previously in validated questionnaires (Ackermann and Driscoll, 2010; Lamontagne and Belanger, 2012). This scale was adopted based on its ability to gather nuanced data on the severity of pain and perceived effectiveness of treatments. The 0 to 10 scale has the added benefit of a definitive 0 point (relevant only for questions that have a definitive 0 answer) and is simple for most people to conceptualize, as similar scales are used in everyday life (e.g., percentage points). For each injury, they were asked to describe the pain using checkboxes (multiple selections allowed) for descriptors “tingling,” “numbness,” “pain,” “weakness,” “cramps,” “involuntary movement,” and “other.” Respondents were asked whether their PRMD had been diagnosed by a healthcare professional, and if so, to specify what the diagnosis was. They were also asked to what extent they had recovered from their PRMD(s) between a choice of “No, not recovered at all,” “Somewhat recovered,” “Mostly recovered,” and “Yes, completely recovered.” A final question asked respondents to rate the effectiveness of various remedial and preventive solutions that they had experience with (multiple selections allowed), on a scale of 0 (no effect) to 10 (greatest effect of all). The list of treatments was adapted from Ackermann and Driscoll (2010). Additional candidate treatments were added to the list based on consultation with numerous oboists and musicians’ health researchers. In these final five questions, respondents were instructed to skip any rows that discussed a PRMD or preventive measure they did not have experience with.

For quality control, the survey was informally pilot tested by performing arts medicine researchers at the University of Toronto and several professional and student oboists in Canada. Reviewers were asked whether the survey’s questions were clear, whether any information should be added or omitted, and whether, in their opinion, the survey would serve to answer the stated research questions. Changes to the survey prompted by these reviewers included changes to wording to increase accessibility of the survey, and the addition of treatments to the final question.

A copy of the questionnaire can be found in the Supplementary Materials.

### Statistical Analysis

Descriptive statistics and associated plots were created in Python using the *pandas* package (Mckinney, 2010) and in Microsoft Excel, respectively. Statistical analyses (e.g., logistic regression analysis) were conducted in R and in Python using the *pandas* and *statsmodels* packages (Seabold and Perktold, 2010). Statistical analyses were performed in regression contexts to assess risk factors associated with PRMDs both categorically (0 = no history of PRMD, 1 = history of PRMD, with models run in the logistic regression framework) and dimensionally (severity of worst PRMD on scale of 1 – 10 analyzed as a continuous outcome utilizing only individuals reporting at least one PRMD, with models run in an ordinary least squares framework). We were interested in five risk factors motivated by literature review (gender, hours of playing, career level, age, years of playing experience). No correction for multiple tests across these five factors was performed. A significance level of p(alpha) < 0.05 was selected for reporting. Additionally, results that survive Bonferroni correction for conducting two analyses within each risk factor (the categorical prevalence model and the two dimensional severity model) are marked in-text with an asterisk (*: *p* < 0.025). Subjects with missing age data were excluded from models that included the age variable. For models that included the gender variable, two gender groups that contained only a single observation were excluded (“non-binary” and “Other”).

## Results

### Prevalence and Severity

Of all respondents, 74.9% (167/223) reported having at least one PRMD in their lifetime. Seventy-nine (47.3%) of these 167 reported having had a diagnosis from a medical professional, while 84 (50.3%) had not received a diagnosis. The remaining 4 (2.4%) individuals were either among the 8 who selected “not applicable,” or they did not respond to this question.

**FIGURE 3:**
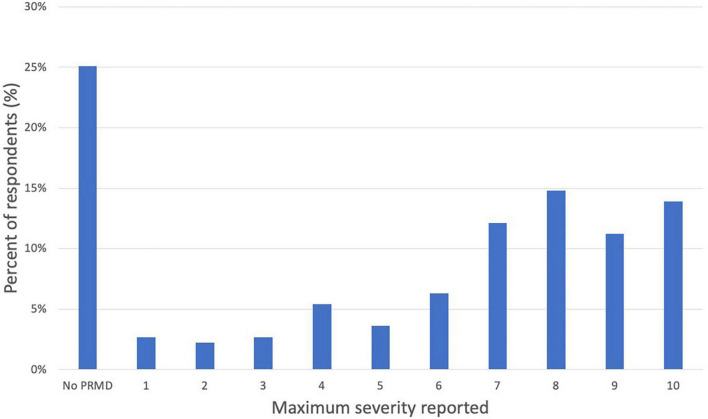
Distribution of maximum severity reported (scale of 1 to 10).

A majority of respondents reported having had severe injuries, with 52.0% (*n* = 116) of all respondents reporting at least one injury rated between 7 and 10, and 61.9% (*n* = 138) reporting at least one injury rated 5 or higher (see Figure 3).

### Location and Symptoms of Injuries

**FIGURE 4:**
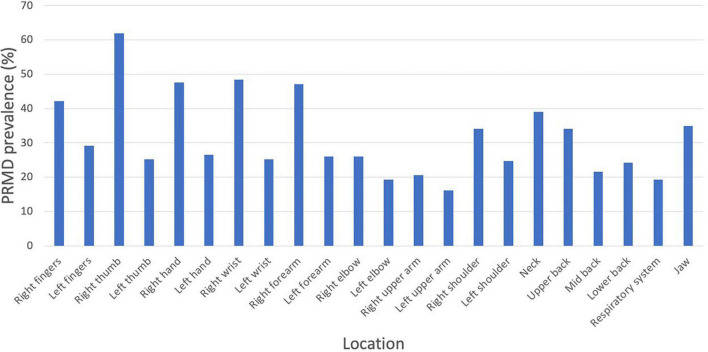
PRMD prevalence (%) by injury location.

**FIGURE 5:**
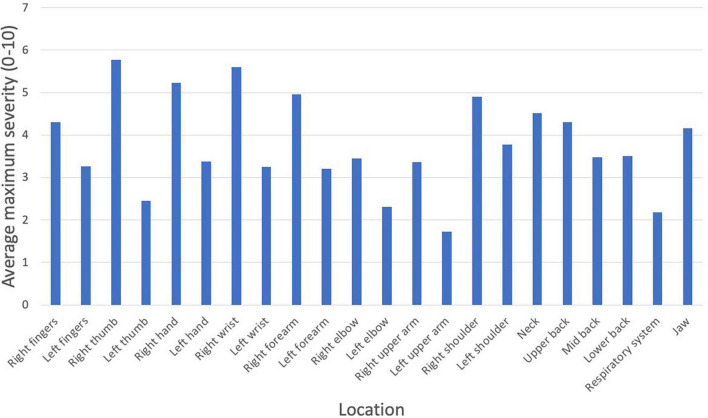
PRMD average maximum severity (scale of 1 to 10) by injury location.

A majority of injuries were on the right side of the body. The most common PRMD location was the right thumb (61.9%), followed by the right wrist (48.4%), right hand (47.8%), and right forearm (47.5%). The most common injuries also tended, on average, to be the most severe: the average severity rating was highest in the right thumb (5.77), right wrist (5.59), right hand (5.23), and right forearm (4.96). See Figures 4, 5 for a complete list of prevalence and average severity by location.

**FIGURE 6:**
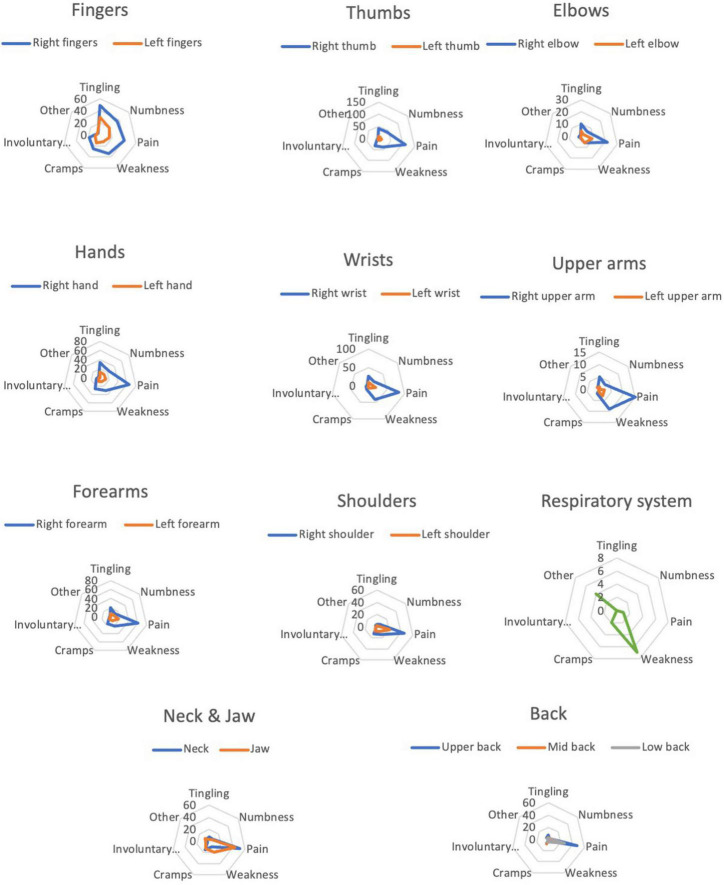
Sensation profiles for each PRMD location.

Pain was the most common descriptor for all injury locations except for respiratory system (weakness) and right and left fingers (tingling). See Figure 6 for complete sensation profiles for all injury locations.

### Recovery Rates

Not all respondents reporting an injury answered the question on recovery. For unknown reasons, a surprising number of participants chose not to disclose recovery status. For example, of 138 people reporting a right thumb injury, only 121 disclosed recovery status. Out of a total of 1,545 injuries reported, recovery data for 1,072 of these injuries was provided (for a response rate on the recovery question of 69.4%). This caveat being said, of those who did respond to this question only 280 of these 1,072 injuries (26.1%) were reported as being fully recovered (see Table 1).

**TABLE 1:** Number of respondents reporting recovery.

Body part	Percent of respondents reporting a lifetime injury	Number of injured respondents who reported on recovery status	Completely recovered (*n*)	Mostly recovered (*n*)	Somewhat recovered (*n*)	Not recovered at all (*n*)
Fingers (right)	42.2% (*n* = 94)	88	28	26	21	13
Fingers (left)	29.1% (*n* = 65)	51	16	15	14	6
Thumb (right)	61.9% (*n* = 138)	121	29	38	35	19
Thumb (left)	25.1% (*n* = 56)	32	12	6	10	4
Hand (right)	47.5% (*n* = 106)	95	26	28	31	10
Hand (left)	26.5% (*n* = 59)	36	13	9	11	3
Wrist (right)	48.4% (*n* = 108)	102	24	35	33	10
Wrist (left)	25.1% (*n* = 56)	36	12	6	15	3
Forearm (right)	47.1% (*n* = 105)	74	19	25	21	9
Forearm (left)	26.0% (*n* = 58)	24	10	3	8	3
Elbow (right)	26.0% (*n* = 58)	29	8	10	8	3
Elbow (left)	19.3% (*n* = 43)	14	4	2	6	2
Upper arm (right)	20.6% (*n* = 46)	26	7	8	8	3
Upper arm (left)	16.1% (*n* = 36)	10	1	2	5	2
Shoulder (right)	34.1% (*n* = 76)	56	13	17	16	10
Shoulder (left)	24.7% (*n* = 55)	31	6	8	9	8
Neck	39.0% (*n* = 87)	62	9	18	24	11
Upper back	34.1% (*n* = 76)	55	7	18	18	12
Mid back	21.5% (*n* = 48)	26	5	7	9	5
Lower back	24.2% (*n* = 54)	34	6	9	12	7
Respiratory system	19.3% (*n* = 43)	12	6	2	1	3
Jaw	35.0% (*n* = 78)	58	19	18	15	6

### Risk Factors

We were interested in a number of variables previously linked to PRMD prevalence and severity (see Introduction). In the following sections, we describe the results of regression analyses quantifying the effects of gender, playing hours, career level, age, and years of playing experience on PRMD prevalence and severity.

#### Gender

See Table 2A for descriptive statistics of PRMD prevalence and maximum severity of PRMD for male and female oboists. *Prevalence model*. Although females were more likely to report a PRMD than males, the difference was not statistically significant. *Severity model*. Females reported a significantly higher severity rating of their worst PRMD (Contrast: 0 = F vs. 1 = M, β = −1.87, *t* = −3.39, *p* < 0.001*).

**TABLE 2:** Descriptive statistics (Gender, Playing hours, and Career level).

	N_noPRMD_	N_PRMD_	%_PRMD_	M_severity_	SD_severity_
**A. Gender**					
Male	19	46	70.8%	4.05	3.44
Female	37	119	76.3%	5.88	3.79
**B. Weekly playing hours**					
1 - 3 h	15	15	50.0%	3.47	4.00
4 - 6 h	9	19	67.9%	4.10	3.84
7 - 9 h	1	36	97.3%	6.86	2.52
10 - 12	8	27	77.1%	5.20	3.59
13 -15 h	5	19	79.2%	5.75	3.59
16 - 18 h	1	14	93.3%	7.19	7.19
19+ h	17	33	66.0%	5.24	5.24
**C. Career Level**					
Student	8	29	78.4%	5.21	3.32
Amateur	16	45	73.8%	4.69	3.61
Professional	32	91	74.0%	5.73	3.94

#### Playing Hours

**FIGURE 7:**
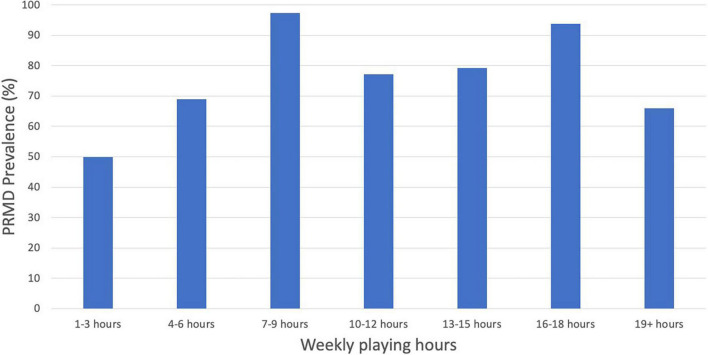
PRMD prevalence (%) by weekly playing hours.

See Table 2B for descriptive statistics of PRMD prevalence and maximum severity of PRMD for each of the seven “hours played per week” categories. There were several significant differences in PRMD prevalence and severity based on number of hours played per week. *Prevalence models*. Figure 7 shows that there is potentially a complex relationship between prevalence of PRMD and hours of playing per week. To test this, we conducted a regression analysis, with the seven evenly spaced categories of “hours of playing per week” coded in orthogonal polynomial form to capture possible complex-shaped relationships with prevalence of PRMD. Coding of the seven categories resulted in six regressors coding for shapes ranging from a simple linear relationship between the two variables through a sixth order polynomial relationship. For regression statistics, see Table 3A. We found (1) a negative quadratic relationship, suggesting an inverted “U” shape relationship*, and (2) a negative fifth degree polynomial relationship*, confirming that the visual observation of multiple peaks in Figure 7 is a statistically significant complex pattern. This regression analysis could alternatively be conceptualized with treatment coding rather than polynomial coding to contrast prevalence of PRMD in the 1 to 3 h of practice per week group to all of the other groups. For regression statistics, see Table 3B. We found PRMD was more prevalent for individuals who played 7-9*, 10-12, 13-15, and 16-18* h per week than for individuals who played only 1 to 3 h per week.

**TABLE 3:** Regression analysis (Playing hours).

	A. Prevalence - Polynomial Coding			
Contrast	Beta	Std. Error	z	p
Linear	0.67	0.54	1.24	0.21
Quadratic	–1.78	0.48	–3.68	0.00*
Cubic	0.41	0.68	0.61	0.54
4th degree	–0.83	0.67	–1.24	0.21
5th degree	–1.99	0.79	–2.53	0.01*
6th degree	0.96	0.66	1.46	0.15

	**B. Prevalence - Treatment Coding (vs. 1 - 3 h)**			
**Contrast**	**Beta**	**Std. Error**	**z**	**p**

4 - 6 h	0.80	0.54	1.47	0.14
7 - 9 h	3.58	1.08	3.33	0.00*
10 - 12 h	1.22	0.54	2.24	**0.03**
13 - 15 h	1.34	0.62	2.15	**0.03**
16 - 18 h	2.71	1.10	2.47	0.01*
19+ h	0.66	0.47	1.41	0.16

	**C. Severity - Polynomial Coding**			
**Contrast**	**Beta**	**Std. Error**	**t**	**p**

Linear	1.96	0.66	2.96	0.00*
Quadratic	–1.65	0.61	–2.69	0.01*
Cubic	–0.08	0.69	–0.12	0.91
4th degree	–0.73	0.73	–1.00	0.32
5th degree	–1.76	0.72	–2.45	0.02*
6th degree	0.86	0.66	1.31	0.19

	**D. Severity - Treatment Coding (vs. 1 - 3)**			
**Contrast**	**Beta**	**Std. Error**	**t**	**p**

4 - 6 h	0.64	0.94	0.68	0.50
7 - 9 h	3.40	0.89	3.82	0.00*
10 - 12 h	1.73	0.90	1.93	0.06
13 - 15 h	2.28	0.99	2.30	0.02*
16 - 18 h	3.72	1.12	3.32	0.00*
19+ h	1.77	0.84	2.12	**0.04**

bold face: p < 0.05; *p < 0.025.

**FIGURE 8:**
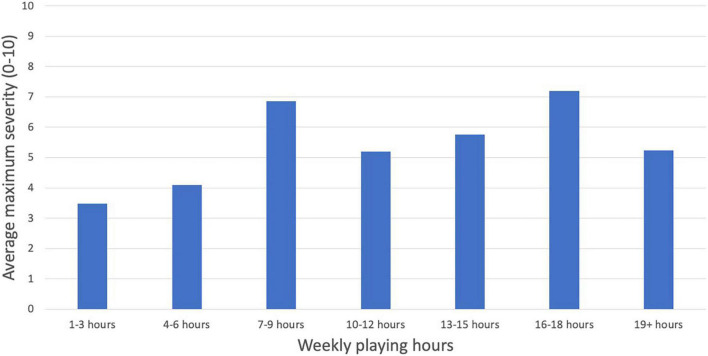
PRMD average maximum severity (scale of 1 to 10) by weekly playing hours.

*Severity models*. Figure 8 shows the complex shape of the relationship between maximum PRMD severity and hours of playing per week; thus we used the same polynomial coding described in the previous section. For regression statistics, see Table 3C. We found (1) a positive linear relationship between the maximum severity of injuries and hours of playing per week*, suggesting that overall longer playing hours was associated with more severe injuries, (2) a negative quadratic relationship*, suggesting an inverted “U” shape relationship, and (3) a negative fifth degree polynomial relationship*, confirming that the visual observation of multiple peaks in Figure 8 is a statistically significant complex pattern. As in the previous section, we reran the regression analysis with treatment coding to contrast severity of PRMD in the 1 to 3 h per week group to all of the other groups. For regression statistics, see Table 3D. We found maximum PRMD severity was significantly higher for individuals who played 7-9*, 13-15*, 16-18*, and 19+ h per week than for individuals who played only 1 to 3 hours per week. The contrast of the 10-12 h per week group and the 1-3 h per week group was trending but not statistically significant.

#### Career Level

See Table 2C for descriptive statistics of PRMD prevalence and maximum severity of PRMD for students, amateurs, and professional oboists. *Prevalence model*. Although student and professional musicians reported higher rates of PRMD than amateurs, no statistically significant group differences in PRMD prevalence were found based on career level. *Severity model*. Although professionals reported the most severe PRMDs, followed by students, then amateurs, there were no significant differences in maximum PRMD severity based on career level.

#### Age

*Prevalence model*. No significant relationship was found between age and prevalence of PRMD. *Severity model*. No significant relationship was found between age and maximum PRMD severity.

#### Years of Playing Experience

*Prevalence model*. No significant relationship was found between years of playing experience (calculated as the difference between current age and the age at which participants began playing the oboe) and prevalence of PRMD. *Severity model*. No significant relationship was found between years of playing experience and maximum PRMD severity.

### Treatment Methods

**FIGURE 9:**
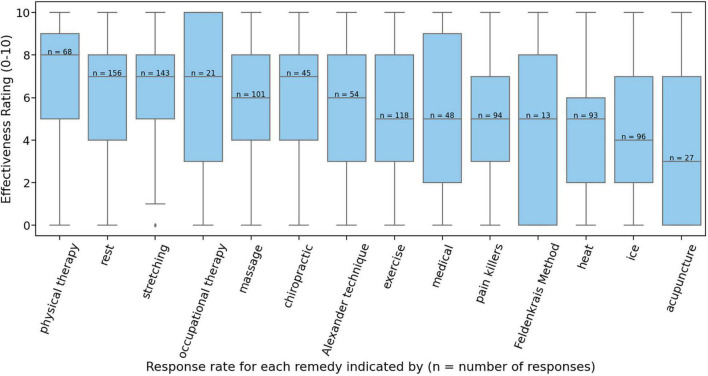
Effectiveness ratings (scale of 0 to 10) of treatment methods.

The treatment methods rated as most effective varied from individual to individual. As not all respondents had tried every method presented in question #10, there was a great deal of variability in the response rates for each method (e.g., 156 respondents evaluated “rest,” while only 13 evaluated “Feldenkrais”). Rest, stretching, exercise, massage, ice, and pain killers had the highest response rates, while Feldenkrais, occupational therapy, acupuncture, chiropractic, medical, and Alexander Technique had the lowest response rates. Despite this variability in response rates, some trends can be discerned. The mean rankings, on a scale of 0 to 10, of physical therapy (6.69), rest (6.37), stretching (6.22), occupational therapy (6.05), and massage (5.94), were higher than most. It is worth noting, however, that for every method there was a very wide variability in perceived effectiveness, and it is clear that there is no single universally effective method (see Figure 9).

Several respondents suggested treatment methods that were not listed in the survey (Table 4). The most common of these was instrument modifications, such as pegs and neck straps that help take weight of the oboe off of the thumb. Body mapping (an awareness technique related to Alexander Technique) and technique or hand position corrections were also mentioned multiple times. Most of these alternative methods were rated very highly, but a few (such as change of activity and marijuana) rated below 5.

**TABLE 4:** “Other” treatment methods responses and ratings.

Method	Effectiveness rating	# of respondents
Instrument modifications (e.g., Fhred, WRIST, neckstrap, special thumb support)	8, 7, 8, NA, 10, 6	6
Body Mapping, body awareness	10, 9, 8,	3
Technique/hand position corrections	8, NA, NA	3
Jaw or embouchure exercises	10, 5	2
Osteopathy	NA, 10	2
Ultrasound therapy on fingers & hands	8, 10	2
Yoga	10, 10	2
Acu-magnetic treatment (related to acupuncture)	8	1
Alternating hot and cold water	7	1
Change of activity	3	1
Compression glove	10	1
Electric impulse therapy	10	1
Energy healing	10	1
Marijuana	4	1
Mindfulness	10	1
Pilates	10	1
SalonPas^®^ pads while sleeping	10	1
Surgery	10	1
Wrist brace	7	1

## Discussion

This study sought to (1) ascertain the prevalence of PRMDs in oboists, (2) determine any predicting demographic factors, and (3) evaluate the self-reported efficacy of various treatment methods in preventing and remedying these injuries in oboe players. The study has the advantage of being the largest sample size of oboists in any known comparable study.

### Prevalence, Severity, and Location of Injuries

Of the 223 survey respondents, 167 (74.9%) reported having had a PRMD in their lifetime. Though there is a wide range of prevalence rates in the literature, this is often attributed to the wide variety of definitions used for PRMD (e.g., Berque et al., 2016). Our result falls in line with results of comparable studies that also used Zaza and Farewell’s definition of PRMD (1997). These studies similarly reported a lifetime PRMD frequency among musicians of between 63 and 78% (Baadjou et al., 2015; Berque et al., 2016; Ballenberger et al., 2018; Kok et al., 2018; Shanoff et al., 2019). Our result is also in the range of oboe-specific PRMD rates previously determined by smaller studies: 75% (with a sample size of 12 oboists), 72% (with a sample size of 60), and 85.7% (with a sample size of 28) (Thrasher and Chesky, 2001; Nemoto and Arino, 2007; Stanek et al., 2017).

By gathering data on injury severity, we were also able to achieve a more nuanced understanding of the degree to which oboists are incapacitated by their injuries. A majority of respondents had experienced severe injuries: 52.0% of all respondents reported at least one injury rated between 7 and 10 out of 10, and 61.9% reported at least one injury rated 5 or higher.

The most common injuries were found to be on the right side of the body, which confirms the findings of previous studies on oboe players (Thrasher and Chesky, 2001; Nemoto and Arino, 2007). The injuries that were the most common (right thumb, right wrist, right hand, and right forearm) also tended to be the most severe. The increased frequency and severity of injuries on the right side of the body can be attributed to the asymmetrical playing posture of oboists, where the right thumb is statically loaded by the weight of the instrument.

### Risk Factors

#### Gender

Numerous studies have found female musicians to have a higher rate of injury than males (Roach et al., 1994; Zetterberg et al., 1998; Steinmetz et al., 2010; Paarup et al., 2011; Ranelli et al., 2011; Nawrocka et al., 2014; Kok et al., 2018). We hypothesized that female oboists would similarly report a higher rate of injury compared to their male counterparts. This study likewise found that females were slightly more likely to report a PRMD than males (76.3% compared to 70.8%), however, this was not a statistically significant effect. We did find, however, that females reported significantly more severe injuries than males. The higher rate and/or severity of PRMD among females is most often attributed to a lack of upper body strength in females compared to males.

#### Playing Hours

The relationship between playing hours and PRMD is complex. Our hypothesis that increased playing hours would contribute to injury was partially confirmed by the linear regressor in our logistic regression analysis - a higher number of hours of oboe playing time per week significantly increases PRMD severity – however there was no similarly statistically significant linear effect on PRMD prevalence. However, we did find a significant negative quadratic effect (inverted “U” shape) for both PRMD prevalence and severity, indicating that the relationship between playing hours and PRMD prevalence and severity was more complex than we had anticipated. In fact, the 1-3 h and 19+ h groups had the least prevalent and the least severe PRMDs on average. This is likely because those who played very few hours per week may not be playing for long enough hours to develop symptoms, while those who do not have symptoms in the first place are less likely to have to limit the time they spend on the oboe, and may therefore play for upwards of 19 h (or alternatively, due to a form of survivorship bias, individuals who are less prone to injury do not have to limit their practice time). Finally, confirming our observation of multiple peaks in Figures 7, 8, we found a significant fifth degree polynomial relationship, suggesting significant fit for a two-peak and three-trough shaped regressor. In fact, those playing 7-9 and 16-18 h per week reported a significantly higher prevalence and severity of injuries than the 1-3 h per week group (see Tables 3B,C). This result speculatively suggests that although longer playing hours can contribute to PRMD frequency and severity (hence the higher rate in the 16-18 h group), the existence of PRMD in the first place may also force musicians to decrease their playing hours in order to manage their injury (explaining why those practicing 7-9 h were also highly likely to report injuries). Because this complex relationship was not *a priori* hypothesized, future work should investigate the relationship between playing hours and injury more thoroughly.

#### Career Level

We hypothesized that the varying demands of different career levels would have an effect on PRMD prevalence and severity, as students and professionals both typically have heavier playing-related demands placed on them than amateurs. Students were found to have a slightly higher rate of injury when compared to professionals and amateurs, while professionals reported more severe injuries than either students or amateurs. However, these effects were not statistically significant.

No known studies have sought to directly compare PRMD prevalence and severity between amateur, student, and professional musicians. This is likely because studies typically target one group or another, and usually end up studying a homogeneous group of students, or professional musicians, or amateurs, but rarely a combination. One study found amateurs to have a one year PRMD rate comparable to the rate established in the literature of professional musicians, but did not also survey professionals to directly compare the effects of career level (Kok et al., 2018). In order to more definitively ascertain the effect of career level on injury in musicians in general, more research is recommended.

#### Age

Previous studies have had mixed results regarding age and musicians’ PRMD. Our study found no significant link between age and PRMD among this sample of oboe players, which echoes the results of a few previous studies (Crnivec, 2004; Rohwer, 2008; Kenny et al., 2018). The few papers that link age with PRMD do not agree on the nature of the relationship: one study found that increased age was associated with increased rates of PRMD (Berque et al., 2016), while another paper found that both the youngest and oldest musicians were more likely to be injured than others, though in their study the youngest and oldest musicians also reported the highest practice times, so the injuries could be related to time spent playing the instrument rather than age (Abreu-Ramos and Micheo, 2007).

#### Years of Playing Experience

Based on several studies that found that players with less experience are more likely to be injured (Yeung et al., 1999; Davies and Mangion, 2002; Ranelli et al., 2011; Nawrocka et al., 2014; Kok et al., 2018; Selms et al., 2020), we hypothesized that fewer years of playing experience would likewise correspond with higher PRMD prevalence and/or severity. However, we found no significant relationship between years of playing experience and injury prevalence or severity.

### Treatment Methods

As not every respondent had experience with all of the treatment methods listed, there was a great deal of variability in the response rate for each method. This response rate variability makes it difficult to meaningfully interpret much of the data. For example, only 13 respondents indicated having had experience with Feldenkrais, while 156 were able to rank the effectiveness of rest. Even for those methods that had higher sample sizes, there was a great deal of variability in perceived effectiveness rankings. There is no strong evidence of any particular therapy being universally beneficial for everyone, as in almost every case there was a group of respondents ranking the effectiveness of the therapy as “0 (no effect).” It is highly likely that the effectiveness of particular methods at treating PRMD is largely dependent on the individual and the particular characteristics of the PRMD at hand. It is worth noting that physical therapy and occupational therapy were among the highest-ranking, and their relative success at alleviating PRMD may be attributed to the more targeted nature of these methods.

Based on the written-in responses in the “other: please specify” section, future studies on treatment method efficacy should consider investigating options such as instrument modifications (e.g., neck strap, instrument pegs), changes to playing technique, and body mapping.

In the literature, there are mixed results in studies exploring the effects of exercise on PRMD prevalence. Some studies have found no association between exercise and PRMD (Brandfonbrener, 1997; Kreutz et al., 2008; Baadjou et al., 2015). Other studies have found increased exercise is associated with less risk of PRMDs (Roach et al., 1994; Yeung et al., 1999; Ranelli et al., 2011). In one study, increased exercise was associated with higher rates of knee pain (Zetterberg et al., 1998). Three studies examined the effect of targeted exercise programs on the rate of PRMD in a group of musicians, and all reported a reduction of injuries at the end of the program (de Greef et al., 2003; Lopez and Martinez, 2013; Chan et al., 2014). All these exercise studies examined a diverse population, consisting of a mixture of string, woodwind, brass, and other musicians. The effect of posture has been explored with sample of saxophone players, and it was found that a rounded upper back and/or backward pelvic tilt correlated strongly with higher pain ratings, and a rounded back, backward pelvic tilt, and excessive curve in the low back all corresponded with higher rates of PRMD in the right wrist (Shanoff et al., 2019). Since the oboe, like the saxophone, has been found to be prone to PRMDs in the right wrist due to static loading of the right thumb (Nemoto and Arino, 2007), it is likely that postural issues will have a similar effect on PRMDs among oboe players.

### Study Strengths and Limitations

An important strength of this study was its ability to reach the largest sample of oboists of any comparable study (*n* = 223). This was aided by the strong online presence of communities of oboists and double reed players. The survey was filled out on a volunteer basis, rather than randomized, and so it is possible that this lack of randomization affected the results, since respondents who have dealt with injuries in the past may be more willing to participate. This study was able to gather valuable information on the perceived effectiveness of various PRMD treatments. However, since the effectiveness of these treatments appears to vary so much based on the individual and the type of injury at hand, it would be advisable for future studies to delve deeper into this issue.

## Conclusion

This study set out to establish the prevalence and nature of PRMD among oboe players, any risk factors that were associated with higher rates of injury, and a ranking of the effectiveness of various treatment methods. We observed the following:

1. The overall lifetime prevalence of PRMDs in this sample of oboists was 74.9%.
2. The most common injuries were on the right side of the body (right thumb, wrist, hand, and forearm were the most common).
3. Of those respondents who provided recovery information about their injuries, only 26.1% of injuries were listed as “completely recovered”.
4. Females reported significantly more severe injuries than males.
5. The relationship between weekly playing hours and PRMD prevalence and severity was complex, with those playing 7-9 and 16-18 h per week reporting significantly more prevalent and severe injuries than those playing 1-3 h per week.
6. While the perceived effectiveness of treatment methods for PRMD varied a great deal between individuals, a few treatments (physical therapy, rest, stretching, occupational therapy, massage) tended to be ranked more highly than others.

With a sample of 223 oboists, this study was able to offer a more nuanced understanding of the nature, prevalence, risk factors, and treatments of PRMD in oboists. The results confirm and expand on much of the existing research on PRMD among woodwind players.

## Data Availability Statement

The raw data supporting the conclusions of this article will be made available by the authors, without undue reservation.

## Ethics Statement

The studies involving human participants were reviewed and approved by University of Toronto Research Ethics Board. The participants provided their written informed consent to participate in this study.

## Author Contributions

HMM conceived of the project, designed and implemented the survey, analyzed data, and wrote and gave critical review of the manuscript. SKL and AER contributed to data analysis. MHT contributed to survey design, analysis plan, and manuscript preparation. All authors reviewed and approved the final version of the manuscript.

## Conflict of Interest

The authors declare that the research was conducted in the absence of any commercial or financial relationships that could be construed as a potential conflict of interest.

## Publisher’s Note

All claims expressed in this article are solely those of the authors and do not necessarily represent those of their affiliated organizations, or those of the publisher, the editors and the reviewers. Any product that may be evaluated in this article, or claim that may be made by its manufacturer, is not guaranteed or endorsed by the publisher.
