# Supplementary material for: Playing-Related Musculoskeletal Disorders, Risk Factors, and Treatment Efficacy in a Large Sample of Oboists
Source: Front Psychol. 2022 Jan 14;12:772357. doi: 10.3389/fpsyg.2021.772357 (PMC8795619; doi:10.3389/fpsyg.2021.772357)
Supplement: Supplementary file 1 [file Data_Sheet_1.pdf]

Oboe Injury Prevention Survey

Background information

1. What is your age?

2. What is your gender?

- Male
- Female
- Non-binary
- Other (please specify)

3. At what age did you begin playing the oboe?

4. Do you consider yourself a student, amateur, or professional oboist?

- Student
- Amateur
- Professional

5. How many hours a week do you spend playing the oboe?

Next

Oboe Injury Prevention Survey

Injury Information

6. A playing-related musculoskeletal disorder (PRMD) is defined as "any pain, weakness, numbness, tingling, or other symptoms that interfere with your ability to play your instrument at the level to which you are accustomed." Have you ever had a PRMD that affected your oboe playing? If so, where? Please rate the severity of any PRMDs you have experienced in each location. Only check locations that apply.

If you have never experienced any PRMD symptoms, please leave all rows blank.

	1 (mild)	2	3	4	5	6	7	8	9	10 (extremely severe)
fingers (right)	○	○	○	○	○	○	○	○	○	○
fingers (left)	○	○	○	○	○	○	○	○	○	○
thumb (right)	○	○	○	○	○	○	○	○	○	○
thumb (left)	○	○	○	○	○	○	○	○	○	○
hand (right)	○	○	○	○	○	○	○	○	○	○
hand (left)	○	○	○	○	○	○	○	○	○	○
wrist (right)	○	○	○	○	○	○	○	○	○	○
wrist (left)	○	○	○	○	○	○	○	○	○	○
forearm (right)	○	○	○	○	○	○	○	○	○	○
forearm (left)	○	○	○	○	○	○	○	○	○	○
elbow (right)	○	○	○	○	○	○	○	○	○	○
elbow (left)	○	○	○	○	○	○	○	○	○	○
upper arm (right)	○	○	○	○	○	○	○	○	○	○
upper arm (left)	○	○	○	○	○	○	○	○	○	○
shoulder (right)	○	○	○	○	○	○	○	○	○	○
shoulder (left)	○	○	○	○	○	○	○	○	○	○
neck	○	○	○	○	○	○	○	○	○	○
upper back	○	○	○	○	○	○	○	○	○	○
mid back	○	○	○	○	○	○	○	○	○	○
lower back	○	○	○	○	○	○	○	○	○	○
respiratory system	○	○	○	○	○	○	○	○	○	○
jaw	○	○	○	○	○	○	○	○	○	○

Other (please specify)

[ ]

7. Thinking about your PRMD(s), how would you describe the sensation? Please select all that apply. Only check locations that apply.

If you have never experienced any PRMD symptoms, please leave all rows blank.

	tingling	numbness	pain	weakness	cramps	involuntary movement	other
Fingers (right)	☐	☐	☐	☐	☐	☐	☐
Fingers (left)	☐	☐	☐	☐	☐	☐	☐
Thumb (right)	☐	☐	☐	☐	☐	☐	☐
Thumb (left)	☐	☐	☐	☐	☐	☐	☐
Hand (right)	☐	☐	☐	☐	☐	☐	☐
Hand (left)	☐	☐	☐	☐	☐	☐	☐
Wrist (right)	☐	☐	☐	☐	☐	☐	☐
Wrist (left)	☐	☐	☐	☐	☐	☐	☐
Forearm (right)	☐	☐	☐	☐	☐	☐	☐
Forearm (left)	☐	☐	☐	☐	☐	☐	☐
Elbow (right)	☐	☐	☐	☐	☐	☐	☐
Elbow (left)	☐	☐	☐	☐	☐	☐	☐
Upper arm (right)	☐	☐	☐	☐	☐	☐	☐
Upper arm (left)	☐	☐	☐	☐	☐	☐	☐
Shoulder (right)	☐	☐	☐	☐	☐	☐	☐
Shoulder (left)	☐	☐	☐	☐	☐	☐	☐
Neck	☐	☐	☐	☐	☐	☐	☐
Upper back	☐	☐	☐	☐	☐	☐	☐
Mid back	☐	☐	☐	☐	☐	☐	☐
Lower back	☐	☐	☐	☐	☐	☐	☐
Respiratory system	☐	☐	☐	☐	☐	☐	☐
Jaw	☐	☐	☐	☐	☐	☐	☐

Other (please specify)

8. Has your PRMD been diagnosed by a health care professional? If yes, what is the diagnosis?

- ◯ Not applicable
- ◯ No
- ◯ Yes (please specify)

9. Have you recovered from your PRMD(s)? How much have you recovered? Only check locations that apply.

If you have never experienced any PRMD symptoms, please leave all rows blank.

	No, not recovered at all	Somewhat recovered	Mostly recovered	Yes, completely recovered
Fingers (right)	○	○	○	○
Fingers (left)	○	○	○	○
Thumb (right)	○	○	○	○
Thumb (left)	○	○	○	○
Hand (right)	○	○	○	○
Hand (left)	○	○	○	○
Wrist (right)	○	○	○	○
Wrist (left)	○	○	○	○
Forearm (right)	○	○	○	○
Forearm (left)	○	○	○	○
Elbow (right)	○	○	○	○
Elbow (left)	○	○	○	○
Upper arm (right)	○	○	○	○
Upper arm (left)	○	○	○	○
Shoulder (right)	○	○	○	○
Shoulder (left)	○	○	○	○
Neck	○	○	○	○
Upper back	○	○	○	○
Mid back	○	○	○	○
Lower back	○	○	○	○
Respiratory system	○	○	○	○
Jaw	○	○	○	○

Other (please specify)

Prev Next

Oboe Injury Prevention Survey

Injury Prevention Methods

10. Below is a list of methods that musicians may use to deal with PRMDs. If you have experience with any of these methods, please rate its effectiveness at alleviating or preventing PRMD symptoms. Only check locations that apply.

	0 (no effect)	1	2	3	4	5	6	7	8	9	10 (greatest effect of all)
rest<br>comments	○	○	○	○	○	○	○	○	○	○	○
ice<br>comments	○	○	○	○	○	○	○	○	○	○	○
heat<br>comments	○	○	○	○	○	○	○	○	○	○	○
exercise<br>comments	○	○	○	○	○	○	○	○	○	○	○
stretching<br>comments	○	○	○	○	○	○	○	○	○	○	○
acupuncture<br>comments	○	○	○	○	○	○	○	○	○	○	○
taking painkillers<br>comments	○	○	○	○	○	○	○	○	○	○	○
medical treatment<br>comments	○	○	○	○	○	○	○	○	○	○	○
physical therapy<br>comments	○	○	○	○	○	○	○	○	○	○	○
occupational therapy<br>comments	○	○	○	○	○	○	○	○	○	○	○
chiropractic<br>comments	○	○	○	○	○	○	○	○	○	○	○
Alexander technique<br>comments	○	○	○	○	○	○	○	○	○	○	○
Feldenkrais<br>comments	○	○	○	○	○	○	○	○	○	○	○
massage<br>comments	○	○	○	○	○	○	○	○	○	○	○
other (please specify)<br>comments	○	○	○	○	○	○	○	○	○	○	○

Prev Done
